# Visual Recognition Software for Binary Classification and Its Application to Spruce Pollen Identification

**DOI:** 10.1371/journal.pone.0148879

**Published:** 2016-02-11

**Authors:** David K. Tcheng, Ashwin K. Nayak, Charless C. Fowlkes, Surangi W. Punyasena

**Affiliations:** 1 Illinois Informatics Institute, University of Illinois, Urbana, Illinois, United States of America; 2 School of Integrative Biology, University of Illinois, Urbana, Illinois, United States of America; 3 Department of Computer Science, University of California Irvine, Irvine, California, United States of America; 4 Department of Plant Biology, School of Integrative Biology, University of Illinois, Urbana, Illinois, United States of America; Penn State University, UNITED STATES

## Abstract

Discriminating between black and white spruce (*Picea mariana* and *Picea glauca*) is a difficult palynological classification problem that, if solved, would provide valuable data for paleoclimate reconstructions. We developed an open-source visual recognition software (ARLO, Automated Recognition with Layered Optimization) capable of differentiating between these two species at an accuracy on par with human experts. The system applies pattern recognition and machine learning to the analysis of pollen images and discovers general-purpose image features, defined by simple features of lines and grids of pixels taken at different dimensions, size, spacing, and resolution. It adapts to a given problem by searching for the most effective combination of both feature representation and learning strategy. This results in a powerful and flexible framework for image classification. We worked with images acquired using an automated slide scanner. We first applied a hash-based “pollen spotting” model to segment pollen grains from the slide background. We next tested ARLO’s ability to reconstruct black to white spruce pollen ratios using artificially constructed slides of known ratios. We then developed a more scalable hash-based method of image analysis that was able to distinguish between the pollen of black and white spruce with an estimated accuracy of 83.61%, comparable to human expert performance. Our results demonstrate the capability of machine learning systems to automate challenging taxonomic classifications in pollen analysis, and our success with simple image representations suggests that our approach is generalizable to many other object recognition problems.

## Introduction

The morphological discrimination of closely related, often congeneric, species is a fundamental problem in pollen analysis, contributing to the low taxonomic resolution of the pollen and spore record [1–4]. A critical example is the discrimination of black and white spruce pollen (*Picea mariana* and *Picea glauca*, respectively), which has challenged researchers since the beginnings of North American palynology [3, 5–8]. Although the morphologies of the pollen grains are similar, the two species have substantial ecological differences. White spruce tends to occupy well-drained upland soils, while black spruce often populates poorly drained lowlands and peatlands [9]. Consequently, their changing abundance in the landscape reflects changing climatic conditions, making these two species of *Picea* important taxa in the reconstruction of North American Late Quaternary paleoclimates [10, 11].

We developed the open-source software ARLO (Automated Recognition with Layered Optimization) to tackle difficult image classification problems such as the identification of black and white spruce pollen. The program applies pattern recognition and machine learning to the identification and counting tasks of pollen analysis. In the field of palynology, analyses are often limited by the rate of data collection [12]. Automation has the potential to exponentially increase palynological sample sizes and increase sample throughput, thereby freeing the palynological researcher to focus on the analysis and interpretation of pollen counts [13]. Larger sample sizes would additionally allow for more accurate estimates of plant diversity, including discovery of rare taxa.

Automated pattern recognition has been a subject of machine learning since its inception in the 1950s [14]. Three general approaches to building a machine learning system for image recognition problems have evolved since that time. These approaches fall along a continuum, based on the amount of background knowledge embedded in the system. On one extreme are purely deductive systems, which are given rules defined by human experts, rather than learnt by machine. These models are often called “expert systems,” to emphasize the amount of expert knowledge needed to develop these systems. In the context of pollen recognition, these rules are derived from image features such as number, shape, and orientation of aperture(s), size, grain shape and symmetry, exine ornamentation, and exine thickness (following [15, 16]). A deductive system would recognize these image features and implement the expert rules. No learning would be involved because the system simply applies pre-defined logic, i.e., following a classification key. At the other extreme are purely inductive systems, where the machine is given no background knowledge (in the form of expert rules or image features) to solve the problem [17]. It is instead only given correctly classified examples (known as the ground truth) for training. The system creates its own rules using the correctly classified examples, by discovering functions that work well at predicting pollen species. Many image analysis systems take some middle ground approach in which an expert specifies image features and the system inductively learns the relative importance of these features in producing correct classifications (e.g. [3, 18]).

The methodology for spruce pollen classification presented in this study is primarily inductive in that it does not involve any problem-specific knowledge in the form of rules or features from human experts. The system is given correctly classified examples of black and white spruce pollen and derives its own features for classification. In this respect, the system follows the behavior of most previous automated pollen classification systems (e.g. [3, 19, 20]). The key difference is the choice of learning system and features used. The features that our system discovers are not the traditional features of pollen morphology that would be recognized by a palynologist. Instead, low-level image-based features, such as simple pixel grid representations, are used. Consequently, our methodology should be applicable to a wide range of visual recognition problems beyond pollen classification. This generalizability, however, comes at a cost. A considerable amount of computation is expended in learning these features from examples.

The two-tier, or two-step, inductive methodology we developed uses machine learning to segment pollen grains from the background slide, classify pollen pixels, and report species ratios. We used ARLO to conduct two separate experiments that utilized two distinct sets of algorithms and optimization procedures. The first analysis was computationally intensive and focused on reconstructing black and white spruce pollen ratios. Instead of segmenting individual pollen grains, we trained and tested our classification model at the slide level. This allowed us to directly compare our automated counts and expert counts to slides of known spruce ratios. In order to achieve results comparable to human experts, we utilized intensive optimization techniques, but our results were not scalable because we used an instance-based machine learning algorithm with *N*^2^ computational complexity (where *N* is the number of examples). This meant that the approach was too computationally expensive to be applied to more than a limited number of samples. Our second analysis focused on maximizing classification accuracy at the individual pollen grain level. Instead of predicting ratios of given slides, we predicted the species represented in a given image window. The resulting analysis was more scalable because we used a hash-based machine learning algorithm whose complexity increased linearly, not quadratically.

In our previous work, we showed that an inductive approach could create machine learning models that accurately mimic expert reconstructions of fossil spruce pollen ratios [3]. The caveat of this approach was that we had no absolute measure of classification accuracy. Our training samples for the previous analysis used examples that were classified by a human expert. However, we had no objective mechanism for verifying the accuracy of the expert identifications. In contrast, for the ratio estimates produced by this study, we expect that our ground truth should have zero error because the pollen slides used for learning (or training) were created by hand from curated pollen samples, with known pollen counts of each species. This nearly eliminates potential error in the training data and allows us to rigorously evaluate our classification system to a greater extent than was possible in our previous work.

## Materials and Methods

### Ethics Statement

Pollen was collected with permission from male cones of trees on private land in Minnesota. To our knowledge, no permits were required for these collections, which complied with all relevant regulations. Neither *Picea mariana* nor *Picea glauca* is a protected species.

### Sample Slide Preparation

Pollen was collected from multiple cones of black spruce (*Picea mariana*) and white spruce (*Picea glauca*) from three stands of spruce trees outside Duluth and Grand Rapids, Minnesota (46.466387°N -92.930358°W; 47.095307°N -93.579061°W; 46.825145°N -93.526411°W) in May 2011. Trees were field-identified by local experts. From this material, we created 12 reference slides (slides containing hundreds of pollen grains from a single individual tree). Two individuals per species were represented; each individual had three replicate slides. These slides comprised a balanced machine learning training set.

We also manually constructed 22 slides with known ratios of black and white spruce pollen to test system performance (Table 1). One hundred individual grains were placed on slides using an eyelash affixed to a dissecting probe. Six unique individuals (individuals that were not represented in the reference set) were used to construct the testing slides, with three individuals per species. The use of unique individuals was to train the system to account for variability among individuals. There is minor variation in pollen shape and size even within pollen originating from a single species or a single cone [21–23], and the use of multiple individuals allowed us to account for the greatest range of morphological variability. Because of this design, the performance of the system was measured, in part, on the identification of pollen from unseen individuals, creating a more challenging recognition problem than has been tested in previous applications of machine learning to pollen identification (e.g. [3, 19, 20]).

**Table 1:** Ratios of constructed slides.

Slide #	Fraction *P*. *mariana*	Fraction *P*. *glauca*
1 and 2	0%	100%
3 and 4	10%	90%
5 and 6	20%	80%
7 and 8	30%	70%
9 and 10	40%	60%
11 and 12	50%	50%
13 and 14	60%	40%
15 and 16	70%	30%
17 and 18	80%	20%
19 and 20	90%	10%
21 and 22	100%	0%

Percentage of *P*. *mariana* and *P*. *glauca* in the 22 manually constructed testing slides. A total of 100 grains was placed on each slide.

### Slide Scanning and Sample Imaging

We used a Hamamatsu NanoZoomer robotic scanning microscope to scan batches of pollen slides at its highest possible optical magnification (400x, 0.23 μm/pixel). This is the standard magnification for pollen analysis using transmitted light microscopy. We manually set the scanning area so that the entire pollen sample on each microscopic slide could be scanned. For each slide, 61 axial (z-) planes were scanned at 1 μm intervals, producing an optically sectioned 3D representation of the entire sample. Uncompressed image data were acquired from the microscope at a rate of about 70 MB/s using the NanoZoomer proprietary raw file format. With cooperation from Hamamatsu, we developed java code to convert the NanoZoomer images into primitive java objects using 2D byte arrays to store band information. Image storage requirements for both the original raw and java-formatted images were about 53 TB in total. We chose to use the uncompressed raw image format because we prioritized the speed of image scanning and processing over storage demands. Due to the size of the raw uncompressed images, we are unable to share the full image dataset online. However, the original reference and constructed ratio slides, raw image scans, and subsampled images are available by request from the corresponding author.

### Computational Work Environment

Our goal in developing ARLO was to build a machine learning framework capable of high- order analysis of biological images. Because our approach is computationally intensive, we took advantage of the supercomputing resources available through the US National Science Foundation’s Extreme Science and Engineering Discovery Environment network (XSEDE). The XSEDE supercomputers have parallel disk IO, so many nodes could simultaneously processes image data. We were granted a 50,000 CPU hour XSEDE startup allocation on the Texas Advanced Computing Center “Lonestar” supercomputer and their next generation supercomputer “Stampede”. Lonestar has 1,888 compute nodes (24 GB memory), 22,656 cores, 44 TB total memory, and 1 PB total disk space. Stampede, the larger machine, has 6,400 compute nodes (32 GB memory), 102,400 cores, 205 TB total memory, and 14 PB total disk space. Several of the slides we processed had a single color band of information that required the larger 32 GB capacity.

### Learning System Overview

**Fig 1:**
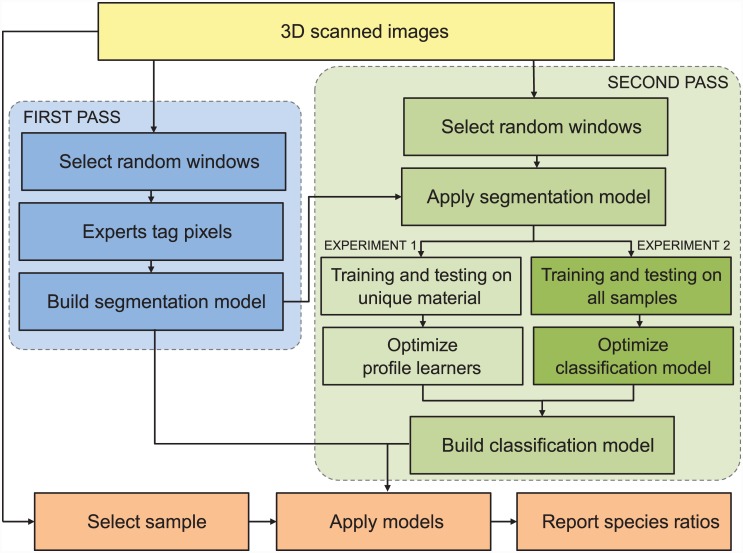
System Overview. Slides were scanned using Hamamatsu’s NanoZoomer microscope. During the first pass, ARLO extracted random image windows that human experts tagged as “pollen” or “not pollen”. Using this information, a segmentation or “Pollen Spotting” model was built to distinguish pollen pixels from the background of an image. During the second pass, ARLO applied this model to a new set of random windows. The pool of samples from which these windows were drawn varied in Experiments 1 and 2. Random windows with enough pollen content were then used to build a pollen species classification model, which was used to predict species ratios in the testing slides in Experiment 1 and to produce species classifications in Experiment 2. See text for details.

ARLO analyzes samples hierarchically in two passes (Fig 1). During the first pass, it identifies important pixels in an image. We call this first step “pollen spotting” because ARLO is effectively distinguishing pollen pixels from background pixels (segmentation of the original image). During the second pass, ARLO classifies these pixels into one of the pre-defined classes supplied by the user. For this paper, ARLO classified these pollen pixels as either black or white spruce. Throughout the process, ARLO optimizes its own control parameters (detailed below), eventually choosing the representation and classification strategy with the highest accuracy solution for the problem. The java code for ARLO is available through SourceForge: http://sourceforge.net/projects/arlo/files/APPS/.

We used ARLO’s two-part analysis to conduct two separate but related classification experiments. The first experiment focused on reconstructing spruce pollen species ratios on known testing slides, given the set of the 12 pure reference (training) slides, and the second on maximizing classification accuracy using all image examples. Both classification analyses, however, rely on the same “pollen spotting” methodology.

### Pollen spotting (Segmentation)

#### Pollen spotting: Training example generation

Ground truth for the identification of pollen pixels from background came from 11 “experts” (student analysts listed in the Acknowledgments) who labeled the same 637 1000 x 1000 pixel windows. These windows were a random subsample of all 30 slides and all 61 z-planes. Windows were of both in-focus and out-of-focus planes. Each image was “segmented” by the experts into pollen and non-pollen pixels by manually marking all pixels that contained pollen. The pixel classifier used the expert labeled pixels (ground truth) to distinguish pollen pixels from background pixels. In most cases, the randomly generated windows did not contain pollen grains, so most examples were of “not pollen” pixels. Our model was trained at the pixel level, and in total there were 7,007,000,000 labeled training examples (pixels). On average, 9% (637,000,000) of the pixels were labeled as “pollen”.

#### Pollen spotting: Algorithm

We used a hash-code based machine-learning algorithm that computes a numeric feature, the “hash code”, an index value for each pixel based on features of its neighborhood [24, 25]. Each hash code indicates the “shape class”, or category, to which the pixel belongs and is an integer ranging from 1 to *N*, where *N* is a system parameter to be optimized. The parameter *N* controls the number of distinct shape classes that the algorithm can recognize. The optimal number of shape classes to learn depends on the complexity of the classification problem and the number of training examples available. For difficult problems, more distinct shape classes are necessary for high accuracy. As more training examples are used, a more complex model (more shape classes) is justified. Constructing hash code examples for learning involves (1) identifying pixels of interest surrounding the pixel to be classified, (2) reducing the resolution of pixel values (quantization), (3) computation of randomly ordered 64-bit hash codes, and (4) reducing the range of hash codes by modulus division.

**Fig 2:**
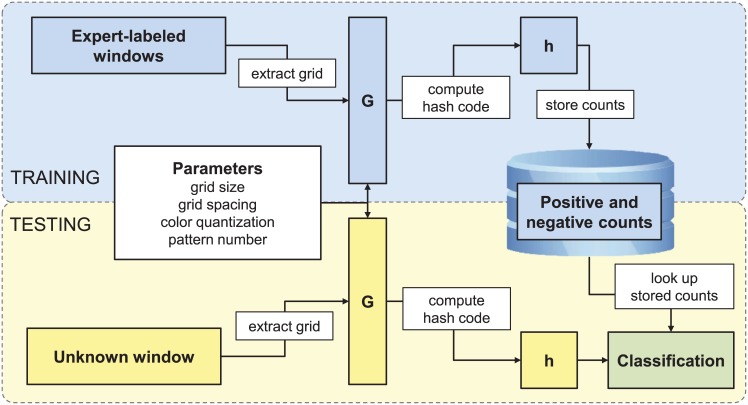
Pixel Classifier Algorithm Overview. During training, grids were extracted from the expert labeled training windows. The input patterns determined the size, scaling, and quantization of these grids. Once grids were extracted, hash codes were computed to reduce dimensionality of the input space. Counts were then stored in a database for both positive and negative examples. During testing, the same process occurred for an unknown image window. Once a hash code was computed for a pixel in the unknown image window, it was looked up in the database of counts to produce a classification. See text for details.

**Fig 3:**
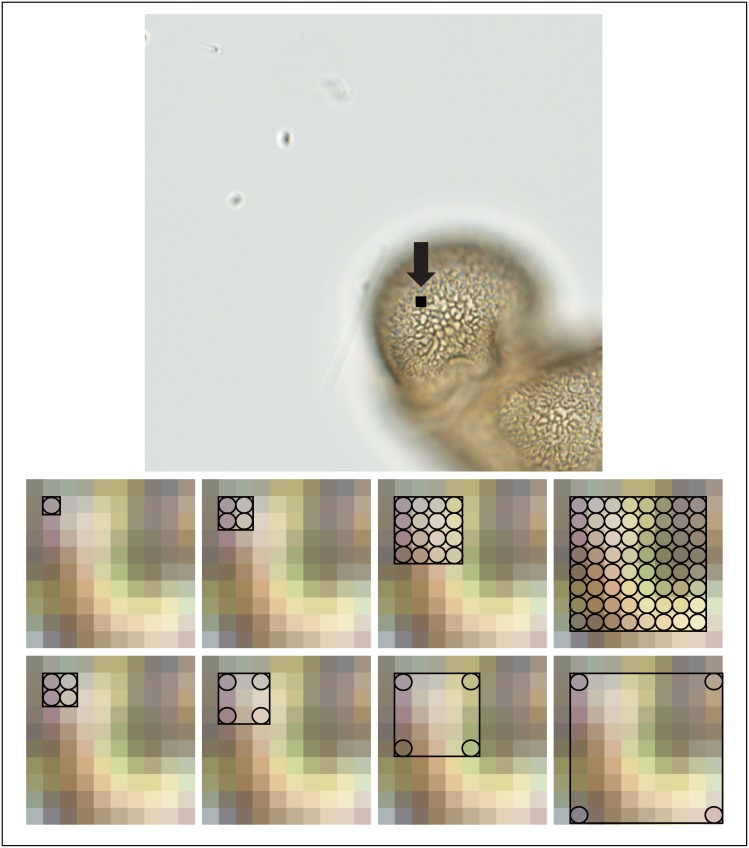
Grid Size and Spacing. At the top is an example of an image window (1000 x 1000 pixels, 0.23 μm/pixel). Highlighted is a 10x10 pixel subwindow. Below is the subwindow with different grid sizes and spacings illustrated. The first row illustrates grid sizes from 1x1 to 2x2, 4x4, and 8x8. A 1x1 grid is a single pixel and only capable of detecting color. Larger grids can detect increasingly complex texture patterns. We explored grids up to 128 x 128 pixels. The third row illustrates a 2x2 grid with different grid spacings (1x, 2x, 4x, 8x) imposed. We explored spacings as large as 128x.

To identify pixels of interest, we looked at every pixel (*p*_*i*_) in every training image and created a “grid example” (*g*_*i*_) for it (Fig 2). The grid example consisted of a grid of pixels centered on the pixel of interest (*p*_*i*_). The number of pixels in the grid and the spacing between them was set by grid size and grid spacing parameters, respectively (Fig 3). Theoretically, the grid size could be as small as 1 x 1 (a single pixel) or as large as 1000 x 1000 (the whole training image). With the smallest grid size, the grid example consisted of only the pixel of interest (*p*_*i*_), but with larger grid sizes, neighboring pixels were included. We considered grid sizes of 2 x 2, 4 x 4, 8 x 8, 16 x 16, 32 x 32, 64 x 64, and 128 x 128. Grid spacing determined the separation between sampled pixels (Fig 3). For example, using an 8 x 8 grid with 1-pixel spacing would mean that all 64 neighboring pixels would be considered. Using an 8 x 8 grid with 2-pixel spacing would mean that every other pixel would be considered. Using an 8 x 8 grid with 128-pixel grid spacing would mean that every 128th pixel would be considered, resulting in an effective size that is larger than all pollen grains of interest. We considered spacings up to 128.

**Fig 4:**
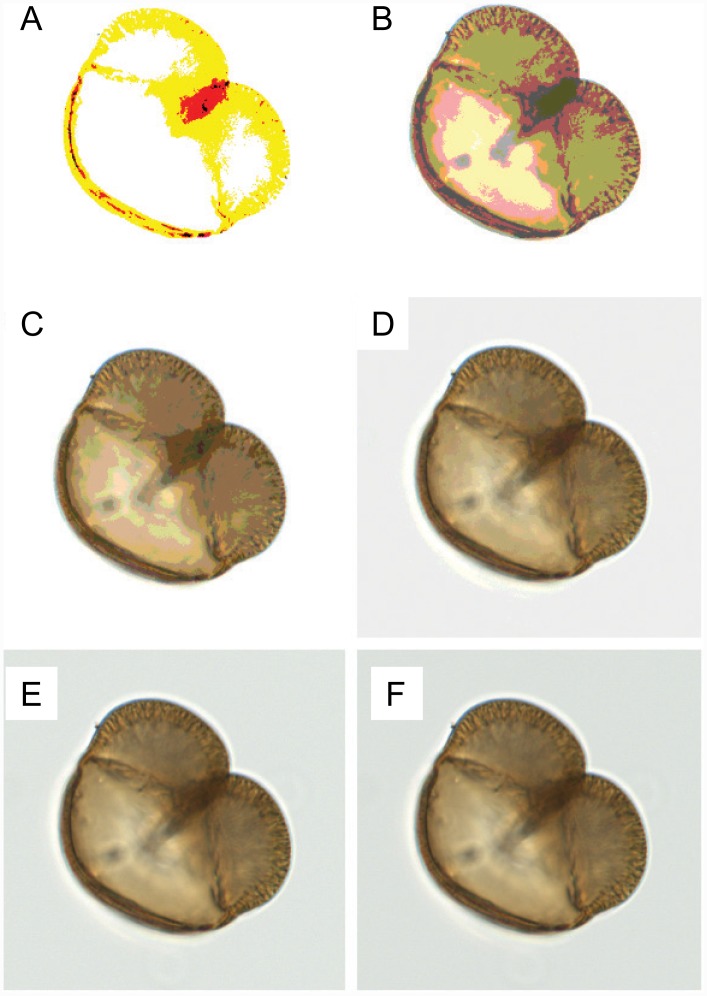
Color Quantization. An image of a black spruce grain is shown with the following color quantizations to reduce the number of possible pixel intensity values: (A) 128x, (B) 64x, (C) 32x, (D) 16x, (E) 8x, and (F) 1x. A color quantization of 128x reduces the possible intensity values to 0 and 1. A color quantization of 1x is the original image. The pollen grain diameter is ~80 μm.

To reduce the number of possible grid examples, we quantized the pixel intensity values. The original pixel intensity values, ranging from 0 to 127, were reduced by a quantization factor between 1 and 127 using integer division, yielding *Q* distinct intensity levels for each color channel (Fig 4). A sampled grid of *N* pixels quantized to *K = Q*^*3*^ possible colors can take on *N*^*K*^ configurations, so even with a small grid and quantization there are still a very large number of possible grid examples. For example a table with *N* = 8 x 8, *K* = 4 x 4 x 4 would have more than 10^115^ combinations.

To reduce the number of combinations to a tractable number, we computed a randomized 64-bit hash code from the grid example. To do this, we used a fixed matrix of random 64-bit numbers of dimensions *N* x *Q*, where *N* is the number of pixels in the grid and *Q* is the number of intensity values. For each of the pixels within a grid example (as defined above), we looked up the associated random numbers in this *N* x *Q* matrix. We then summed the random numbers associated with all the pixels in a grid example to produce a 64-bit hash code. The final hash code *(h*_*i*_) is derived from the 64-bit hash code using modulus division by a factor of *B*, the number of counting bins for accumulating pixel class statistics. In this experiment, *B* = 2,048,000. This means we were tracking about 2 million distinct shape classes and their positive and negative associations with the target class (pollen or not pollen).

Once the hash code *h*_*i*_ was computed, it was used as an index in two arrays containing pattern counts for positive and negative examples (Fig 2). During training, if a human expert classified the pixel *p*_*i*_ from which *h*_*i*_ was computed as a positive example of pollen, the value of the positive pattern count array at the computed index was incremented. Otherwise, the value of the negative pattern count array at the computed index was incremented.

After training was complete, the pattern count arrays, random number tables, and global parameters contained all the needed information to classify new examples and comprised the machine learning “model”. During testing, a grid example was formed from the candidate pixel to be classified using the same parameters used for training (Fig 2). A hash code was then calculated using the same random number tables, and the values corresponding to its index were looked up in both pattern count arrays. If the value of the positive count array was greater than the value of the negative count array, the candidate pixel was classified as a positive example, i.e., as pollen. Otherwise, it was classified as a negative example, i.e., as the slide background. Storing the pattern count arrays and random number tables for a typical set of parameters took about 32 MB of disk space.

This approach is analogous to performing classification with a standard histogram estimator, where the input space is divided into bins and the prediction of a new example is based on the bin into which it falls [26]. However, because the space of grid examples is very high dimensional, it is necessary to use quantization and hashing to reduce dimensionality before building the histogram. Our approach is also closely related to methods for image retrieval and approximates nearest-neighbor search using locality-sensitive hashing [24, 25].

#### Pollen spotting: Optimization

Optimizing the parameters used to construct grid examples directly affects the types of patterns the pixel classifier detected. Grid spacing determines the scale of patterns detected. Grid size and color quantization determine the number of possible hash codes, which affects the complexity of patterns detected. Tuning the model’s scale and complexity was important to achieving high accuracy. We used an automated approach to tune these model parameters based on cross-validation. For a given set of parameters, we generated a model from training examples and tested performance of the resulting system on some validation data. By searching over settings of the parameters, the system can automatically estimate the setting of the parameters that would achieve the highest accuracy identifications on new, unseen test images.

To limit search, control parameters were optimized a single dimension at a time. This was primarily to limit the computational demands of the optimization. For example, we started with a grid size of 1x1, a grid spacing of 1x, and a color quantization of 1x. Keeping the other two parameters constant, we increased grid size from 1 x 1 to 2 x 2, and then from 2 x 2 to 4 x 4, and so on. Accuracy was measured using leave-one-out cross-validation at the training window level, where the system trained on 636 of the 637 training windows and was tested on the one omitted window. This was repeated for all 637 training windows resulting in a 637-fold cross validation. Accuracy was averaged across all 637 iterations. We used the parameter values that yielded the highest accuracy to form the final classification model using all training examples.

### Experiment 1: Reconstructing species ratios

The goal of our first classification experiment was to predict species ratios of the testing slides given only the training (pure reference) slides. This meant that training was accomplished on pollen material from individuals that were not represented in the testing set. Given the potential morphological variation in pollen from different individuals and samples due to both biological and processing variability [21–23, 27–29] this made the training process more challenging than most previously reported machine learning pollen experiments.

#### Species ratios: Training example generation

Instead of using grids of pixels to train our classifier as we did in pollen spotting, in this experiment, we used lines of pixels. In our previous work, we had found that a simplified version of these pixel lines was the most predictive in discriminating between two spruce species [3]. Our approach was based on a single type of feature, which we called a “profile”. A profile was a line of pixels that satisfied two constraints: (1) it had been identified as containing all pollen pixels by our pollen spotting classifier and (2) it had been identified as having information content greater than a given threshold. We define information content (*IC*) as:

$$IC=\;\frac{1}{n}\overset{n}{\underset{i=1}{\sum }}\left(\left|x_{i}-x_{i+1}\right|\right)$$ (1)

where *n* = number of pixels in a profile– 1 and *x*_*i*_ = pixel intensity value of pixel at position *i* of profile *x*. This metric correlates positively with images that are in focus or have a high contrast. The rationale was to focus the machine’s attention on the most informative regions of the images. Therefore, if the information content of a profile was greater than or equal to a threshold of 5.0 pixel intensity units per pixel, the profile was considered high contrast (or in focus). These selected profiles were used as training examples for classification.

For the experiments reported here, we used a profile length of 128 pixels. To reduce computational complexity and algorithm running times, we did the following: (1) only the red color band of each pixel was used (not all three RGB colors), (2) only vertical pixel lines were considered (not other orientations), and (3) only every 5th z-plane was considered for training and testing. Within a z-plane, all pixels were considered. The selection of the red band was arbitrary. In our previous work, we used vertical, horizontal, and diagonal pixel lines read both forwards and backwards [3]. Given the pixel layout in memory, however, it was most efficient to read vertical pixel lines. Limiting the analysis to every 5th z-plane reduced the system run time by a factor of 5. We would expect even higher system accuracy if all color bands, line orientations, and z-planes were analyzed, but we were limited by supercomputer CPU time. Training examples were generated only from the 12 training slides. The process generated 205,407,234 training examples in total.

#### Species ratios: Algorithm

In order to learn from the training examples, the pollen species classifier first had to determine which profiles in the training set were the most effective at discriminating between the pollen of black and white spruce. To accomplish this, profiles were randomly selected one by one from the training set and evaluated. For explanatory purposes, this selected profile can be thought of as a candidate “rule” that can be used to classify other profiles by measuring how close they are to the “rule” profile. For a candidate rule, the Manhattan distance between the rule profile and each profile in the training set was calculated. The profiles that were closer to the learner than a certain threshold (known as the “near-far” threshold, optimization described below) were considered to be “near” profiles, and the profiles that were farther were considered to be “far” profiles. The prediction made by the rule is simply based on the proportions of training examples for each species that fell in the near or far bins.

To evaluate the quality of a candidate rule, each training example was classified using the candidate rule. For example, if a training example was designated as “near” and the baseline probability of being both “near” and black spruce was greater than the baseline probability of being both “near” and white spruce, then the training example was classified as black spruce. After classification of all training examples, the error rate of the rule was determined by comparing the training examples’ assigned class to its actual class. This entire process was repeated with every new rule that was selected. Rules with low error rates had large differences in the class distributions of their “near” and “far” categories and were hence capable of discriminating between the two classes. Once a large set of rules was evaluated, the ones with the lowest error rates were combined with equal weighting to make pollen species classifications and determine slide ratios.

#### Species ratios: Optimization

Model accuracy was dependent on several parameters that we varied to maximize performance. They included the following: (1) pixel line length, (2) “info-content” threshold, (3) “near-far” threshold, and (4) number of learner profiles used to make final classifications.

Pixel line length was optimized by starting with a length of 2, and increasing by powers of 2 until the highest classification accuracy was found. The “info-content” of a pixel line was measured by computing the average of the absolute values of differences between adjacent pixel intensity values. The “info-content” threshold was qualitatively determined by viewing slide images with different thresholds. Because we applied the optimized “info-content” threshold to all images prior to example generation, only the in-focus regions of pollen grains were used for classification. The “near-far” threshold was optimized for each learner profile, by searching for the threshold between 1 and the largest possible distance between two profiles that maximized the info-content of the image. If the pixel line length was 2, the maximum distance between two profiles was 510 pixel intensity units (twice 255, the maximum possible distance between any 2 pixel values in the 8-bit image format). If the pixel line length was 128, the maximum distance between two profiles was 32,640 pixel intensity units. To determine the optimal number of learner profiles to use for classification, the learners were rank-ordered based on their error rates. The top learner was first used on the testing data, then the top 2 learners, then the top 3, and so on, until the optimal number of top performing profiles was determined. The classifications made by these learners were weighted equally.

### Experiment 2: Scalable grain-level species classification

The goal of our second classification experiment was to maximize classification accuracy using a more scalable approach. Instead of using line profiles, during the second pass, the original pixel-based pollen spotting algorithm (as described above in “Pollen spotting”) was applied to the pixels classified as pollen from the first pass. This approach was more scalable than utilizing the line profile classifier used in Experiment 1, which has a more intensive training and optimization process.

#### Grain-level classification: Training example generation

During the second pass (Fig 1), random 256 x 256 windows were sampled from 10 of the 12 pure training slides (100% black spruce or 100% white spruce). A total of 1,232 windows per slide were sampled. Because the windows came from pure training slides, species classifications were already known and there was no need for expert input. The pollen spotting results from the first pass were used to select windows for training. Only those windows that had greater than 50% pollen content were selected as training examples.

#### Grain-level classification: Algorithm

The pixel classifier was trained as described in the section on “pollen spotting,” except in this case, grid examples were only created for pixels labeled as pollen. We only sampled windows from our reference slides, from which an image would be 100% black or white spruce. This was done so we were 100% certain of the true pollen grain class. During testing, 100 random windows were drawn from the two pure training slides that were left out during training. The pixel classifier was applied to the pollen pixels in these windows but allowed to ignore windows it was not confident enough to classify. Confidence was defined as the difference in ratio between the black and white spruce pixels it classified. For example, if the pixel classifier classified a window as containing 100% black spruce pixels and 0% white spruce pixels, its confidence would be 100%. If it predicted 50% black spruce pixels and 50% white spruce pixels, its confidence would be 0%. If the pixel classifier’s confidence for a particular window was lower than the confidence threshold, it did not classify the window. To prevent the classifier from ignoring all windows on a slide, a parameter was used to set the minimum number of windows to classify per slide. This parameter was also determined through optimization.

#### Grain-level classification: Optimization

Optimization was similar to the process used during the first pass (“pollen spotting”), with two modifications. First, two additional parameters for optimization were included: minimum confidence and minimum number of classifications per slide. Also, to increase computational speed, the parameter space was sampled randomly in each iteration, instead of systematically searching the entire parameter space by powers of 2. We had enough computation resources to generate and test 397 randomly chosen parameter points. Each point was tested by measuring the resulting system predictive accuracy, and the highest-scoring parameter point was used for the final model. Accuracy was measured based on cross-validation at the slide level, where the system trained on images from 10 of the 12 training slides and was tested on the remaining 2 slides. This process was repeated for 10 randomly sampled sets of 10 training slides.

## Results

### Pollen Spotting

**Fig 5:**
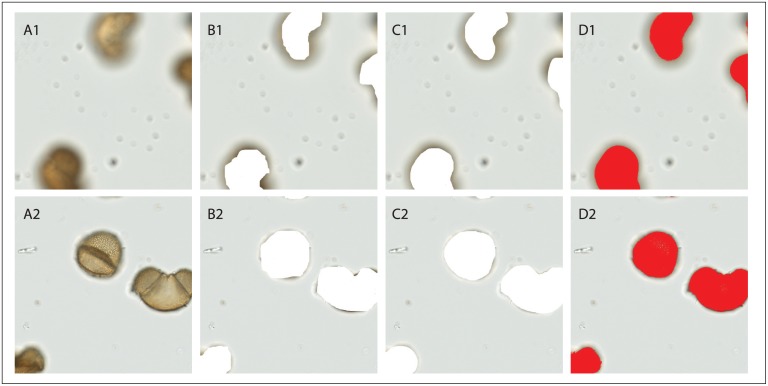
Pollen Spotting Results. Column (A) shows two randomly selected training images with pollen present, taken from the original scanned data set. Each image is 1000 x 1000 pixels, 0.23 μm/pixel. The top image (A1) is out of focus and the bottom image (A2) is in focus. Columns (B) and (C) show the result of expert editing on the windows shown in (A). Two different experts marked the pollen pixels by erasing them from the original image. Column (D) shows pollen pixel predictions (in red) made by the pixel classifier after it had been trained on the 637 training examples provided by each of the 11 experts.

Although human experts agreed at a gross level on what was and was not pollen within an image window, the fine edges of the marked pixel regions differed. Some experts were more conservative and some more liberal in marking out of focus pixels, thus introducing variability in the training set (Fig 5B1, 5B2, 5C1 and 5C2). However, because the majority of pixels viewed were not pollen, on average experts agreed 99.68% of the time at the pixel level.

The machine pollen spotting results more closely followed the contours of the pollen grains (Fig 5D1 and 5D2). Performance of the pixel classifier was a function of the number of training examples. Approximately 500 training windows, classified by all experts, were required to achieve expert level performance. Using all 637 training examples, we produced our most accurate model, which agreed with the human expert pixel-level classifications 99.77% of the time. The most experienced of the experts (as determined by years of experience with pollen analysis) agreed with the other experts 99.76% of the time. Our most accurate model had a grid size of 8 x 8 pixels, a grid spacing of 8 pixels, and a color quantization of 10.

### Experiment 1: Pollen Ratio Results

**Fig 6:**
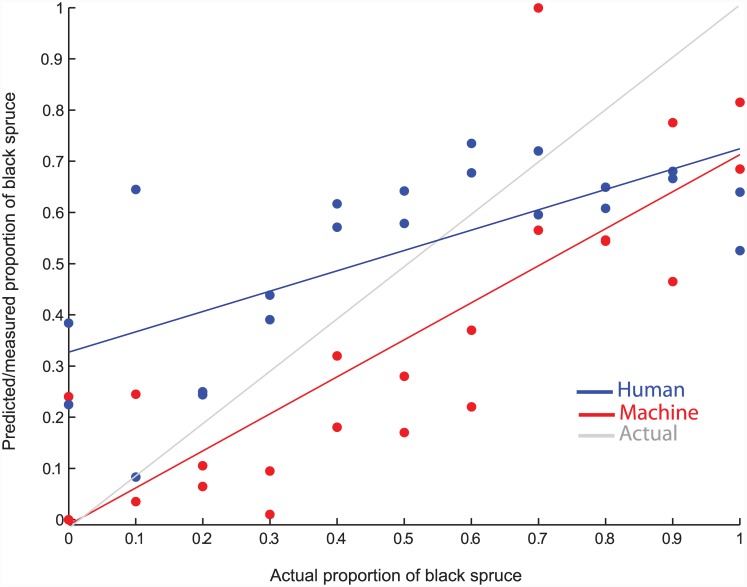
Pollen Ratio Results. Performance of the machine classifier from the first experiment (red) and human expert (blue) in reconstructing the proportion of black spruce pollen from the manually constructed testing slides (Table 1) and their respective lines of least square regression. The actual pollen ratio line is illustrated in gray.

For a baseline comparison of species classification results, we had performance data from one human expert. To measure performance for both the human expert and the machine (the results from the single learner profile with the highest accuracy), we computed a linear regression between predicted and actual fractions of black spruce and used the coefficient of determination (*r*^*2*^) from least squares regression as a measure of accuracy (Fig 6). The machine (*r*^*2*^ = 0.67) outperformed the human expert (*r*^*2*^ = 0.49). However, these results for the machine learner are highly optimistic, given that the model is overfit because the profile was optimized using the testing data.

**Fig 7:**
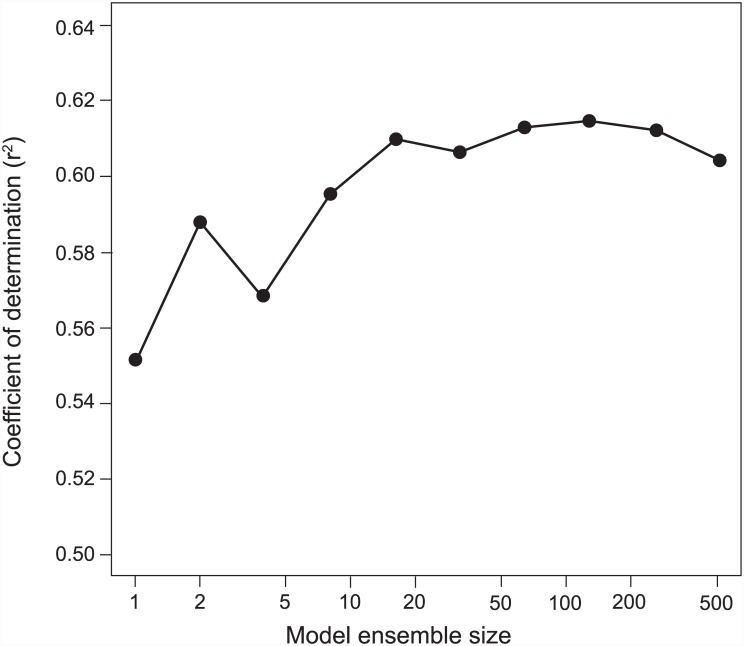
Profile Number versus Predictive Performance. This graph shows the predictive accuracy of the machine learning models on the test slides using the top 1, 2, 4, 8, 16, 32, 64, 128, 256, and 512 profiles in the model ensemble. Results are from the first experiment. 91,696 patterns were tested. The highest accuracy was achieved at around 128 profiles.

When we trained on examples from the training reference slides and measured the performance of the learner profiles relative to only the testing examples, a performance of *r*^*2*^ = 0.55 was achieved using the rule that scored the highest accuracy on the training data. When we combined the top 128 rules relative to the training examples, we achieved a performance of *r*^*2*^ = 0.61 on the test examples. Compared to human expert performance, all rule ensembles, from smallest to largest, performed better. However, it appeared that machine performance suffered as more poor-quality rules were added to the ensemble (performance dropped from 128 rules to 512 rules) (Fig 7). The pixel line length that yielded the highest classification accuracy was 128 pixels. The highest accuracy info-content threshold was 5.0 pixel intensity units per pixel.

### Experiment 2: Grain-level Species Classification Results

**Fig 8:**
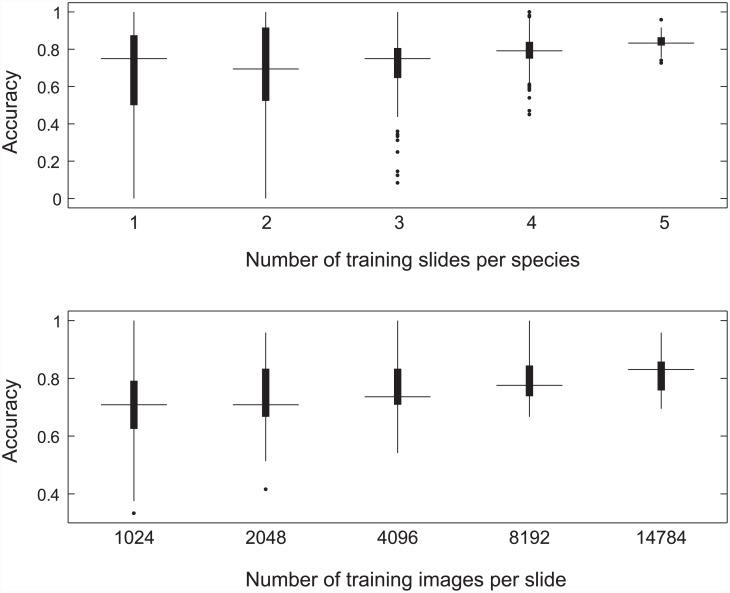
Learning Curves. Box plots summarizing the effect of number of training slides (top) and number of training images per training slide (bottom) on species classification accuracy from the second experiment. Median values are designated by horizontal bars. The edges of the box represent the 25th and 75th percentiles. Whiskers represent ±2.7 SD, or 99% of the data. Outliers are plotted as single points.

The highest accuracy model used by the pixel classifier during the second pass had a grid size of 3 x 3, a grid spacing of 10, a color quantization of 14.24, a minimum confidence of 43.61% and a minimum number of classifications per slide set to 1. This model had an estimated accuracy of 85.85%. This accuracy was suspected to be optimistic, however, because it was based on an average of only 10 cross-validation trials. To estimate the true accuracy of the model, we averaged the results of 271 cross-validation trials using the highest-accuracy parameter point found by the 10 cross-validation trials, which gave us a slide classification accuracy of 83.61%. This machine accuracy compared positively with human expert performance; our human expert averaged 63.9% accuracy on the four pure testing slides (100% *P*. *mariana* or 100% *P*. *glauca*). To estimate the amount of training examples necessary to achieve expert level performance, we computed learning curves. This illustrated how system accuracy changed as a function of the number of pure training slides and the number of training images drawn per slide (Fig 8). With 5 training reference slides and 14,784 training images, the system was able to consistently outperform our human expert.

## Discussion

Our results demonstrate ARLO’s ability to solve a difficult pollen species classification problem using an adaptive machine learning approach. Decomposing the image classification problem into a foreground/background pixel classification problem and a separate species classification problem proved effective. Because ARLO uses a general representation (a pixel grid or pixel line) and parameter optimization to adapt this representation to the given problem complexity and number of training examples, the methodology described in our study should be easily generalizable.

The results of our first experiment indicate that ARLO is capable of training on standard reference specimen slides, which represent data that can be derived without a high degree of expertise. In our experiments, only low-level expertise was required to distinguish between pollen and non-pollen pixels on an image; with some training, undergraduate trainees could do this reliably. Human experts were not used to provide “ground truth” for training the species classifier. This potentially makes the collection of large training datasets rapid and highly efficient. The first experiment also demonstrated that this training can be applied to the classification of pollen specimens from unique individuals. These training samples were drawn from individuals that were not represented in the testing samples, demonstrating ARLO’s ability to tolerate morphological variability that may have been unique to an individual. This again allows for an automated approach to pollen classification that does not require significant training and input from human experts.

The caveat to ARLO’s data-driven approach is that it requires a large amount of data and image processing. For grain-level classification distinguishing between black and white spruce pollen (Experiment 2), the system required 14,784 classified training images. Using expert-defined features potentially could have resulted in a system that needed fewer training images to achieve the same level of accuracy, but would have required more expert input into the labeling of training data.

The features that our system discovers are not the traditional features of pollen morphology that would be recognized by a palynologist. Instead, low-level image-based features, such as simple pixel grid representations, are used. One aspect of this pixel representation is color. The system uses a normalized representation of color and decides how much influence color has on the analysis. Where color is not informative, it does not hold significant weight.

We recognize that our interpretation of the efficacy of our pollen spotting results is limited by our decision to individually vary each variable during optimization. This decision was a pragmatic one, given the computational demands of the analysis. The degree to which this affects our results, however, is arguable. Because we began with values falling within the middle of the range of possible settings, we do not anticipate that further optimization would affect performance significantly. Additionally, the algorithm’s performance depends on the parameters that are discrete, so it is not possible to use gradient-based [30] and other smoothly varying methods for optimizing the parameters. Given these constraints, our use of a coordinate descent approach falls within the norms of standard practice [31].

The discrimination of black and white spruce pollen represents a challenging problem to even the expert palynologist. This is evident in the relatively low accuracy of the expert counts (Fig 6). We recognize that the conditions under which our expert baseline counts were produced were somewhat artificial: there was a limited number of pollen grains on a slide (100 grains) and all grains had to be counted, regardless of orientation and quality of the grain. Under standard counting practices, human analysts would have the benefit of evaluating a far larger number of grains. However, it is notable that the bias of the expert was to overestimate the occurrence of the rare species (Fig 6). Although the overall absolute grain-to-grain accuracy of the human expert was higher for some samples, the machine results better paralleled the overall trend in changes of the ratio of black to white spruce. Capturing trends in relative pollen abundances is arguably the more critical result for paleoecological interpretations [3].

ARLO represents progress toward the problem of automated pollen identification, but it is not a complete solution. Expanding on the results of this work will require experimentation with other more powerful machine learning techniques, including neural networks (NN) and support vector machines (SVM). However, the tradeoff is speed. ARLO represents a very fast recognition algorithm whose power lies in the ability to process large amounts of data. Our choice of image representation was extreme, with every pixel used as an example. (The decision to classify at the pixel-level, however, allowed us to circumvent the need to directly define objects and provided us with the largest possible number of examples). The use of high-resolution images required fast, computationally efficient algorithms. Scaling the analysis to multiple classes of identification will require further experimentation addressing the tradeoffs between speed, accuracy, and computational intensity.

There is a significant need in the biological sciences beyond the field of palynology to standardize and automate the interpretation of images, as our ability to produce high-resolution, high-throughput images is rapidly exceeding experts’ abilities to analyze them. (The number of these experts may also be declining). Our results emphasize the degree to which visually abstract features can be used to solve specific image-based classification problems. The power of this abstraction is that it reduces the complexity of the machine analysis. Features do not need to be known *a priori*. As our image datasets grow, there will be a larger role for efficient algorithms for visual analysis in biological classification.
